# The Growth and Survival of *Mycobacterium smegmatis* Is Enhanced by Co-Metabolism of Atmospheric H_2_

**DOI:** 10.1371/journal.pone.0103034

**Published:** 2014-07-24

**Authors:** Chris Greening, Silas G. Villas-Bôas, Jennifer R. Robson, Michael Berney, Gregory M. Cook

**Affiliations:** 1 University of Otago, Department of Microbiology and Immunology, Dunedin, New Zealand; 2 University of Auckland, The Centre for Microbial Innovation, Auckland, New Zealand; 3 Albert Einstein College of Medicine, Department of Microbiology and Immunology, Bronx, New York, United States of America; University of Padova, Medical School, Italy

## Abstract

The soil bacterium *Mycobacterium smegmatis* is able to scavenge the trace concentrations of H_2_ present in the atmosphere, but the physiological function and importance of this activity is not understood. We have shown that atmospheric H_2_ oxidation in this organism depends on two phylogenetically and kinetically distinct high-affinity hydrogenases, Hyd1 (MSMEG_2262-2263) and Hyd2 (MSMEG_2720-2719). In this study, we explored the effect of deleting Hyd2 on cellular physiology by comparing the viability, energetics, transcriptomes, and metabolomes of wild-type vs. Δ*hyd*2 cells. The long-term survival of the Δ*hyd2* mutant was significantly reduced compared to the wild-type. The mutant additionally grew less efficiently in a range of conditions, most notably during metabolism of short-chain fatty acids; there was a twofold reduction in growth rate and growth yield of the Δ*hyd*2 strain when acetate served as the sole carbon source. Hyd1 compensated for loss of Hyd2 when cells were grown in a high H_2_ atmosphere. Analysis of cellular parameters showed that Hyd2 was not necessary to generate the membrane potential, maintain intracellular pH homeostasis, or sustain redox balance. However, microarray analysis indicated that Δ*hyd*2 cells were starved for reductant and compensated by rewiring central metabolism; transcripts encoding proteins responsible for oxidative decarboxylation pathways, the urea cycle, and ABC transporter-mediated import were significantly more abundant in the Δ*hyd2* mutant. Metabolome profiling consistently revealed an increase in intracellular amino acids in the Δ*hyd*2 mutant. We propose that atmospheric H_2_ oxidation has two major roles in mycobacterial cells: to generate reductant during mixotrophic growth and to sustain the respiratory chain during dormancy.

## Introduction

In recent years, it has emerged that a number of soil *Actinobacteria* of the genera *Mycobacterium*, *Streptomyces*, and *Rhodococcus* oxidise the trace concentrations of H_2_ found in the lower atmosphere [1], [2], [3], [4]. In addition to being biogeochemically important [5], scavenging of tropospheric H_2_ is physiologically unusual; all other characterised hydrogen-oxidising organisms are only capable of recycling the high concentrations of H_2_ evolved through other biological processes or geothermal activity [6]. The purpose and importance of hydrogen scavenging in the physiology of *Actinobacteria* nevertheless remains to be understood. It is also to be determined whether this process influences the composition of microorganisms in soil ecosystems.

Work in our laboratory has resolved the determinants of hydrogen scavenging. The soil bacterium *Mycobacterium smegmatis* catalyses atmospheric H_2_ oxidation using two high-affinity, membrane-associated, oxygen-dependent [NiFe]-hydrogenases [3]. Both of these enzymes are expressed during exponential growth, though their expression and activity is significantly higher during the transition to stationary phase due to carbon-limitation. The fast-acting Group 2a [NiFe]-hydrogenase Hyd1 (MSMEG_2262-2263) is responsible for the majority of whole-cell H_2_ oxidation. In contrast, the Group 5 [NiFe]-hydrogenase Hyd2 (MSMEG_2720-2719) is a much slower-acting enzyme in whole-cells [7], [3]. Despite its low activity, Hyd2 has been shown to be important for the growth of *M. smegmatis* [8]. Furthermore, orthologs of this enzyme are more widely distributed among sequenced *Actinobacteria* and are apparently responsible for the tropopheric H_2_ uptake of streptomycetes and rhodococci [9], [4]. It should also be noted that *M. smegmatis* also encodes a further hydrogenase, Hyd3; this enzyme is only expressed during oxygen-limitation, where we propose it serves to couple the reoxidation of NAD(P)H to the evolution of hydrogen [7], [8].

In this work, we provide insight into the physiological role of hydrogen scavenging by observing the effect of deleting Hyd2 throughout exponential growth, upon entry into stationary phase, and during long-term survival. Using a combinatorial approach, we show that hydrogen scavenging is required for the efficient metabolism of certain carbon sources and infer that atmospheric H_2_ is a source of reductant for mycobacterial metabolism.

## Materials and Methods

### Bacterial strains and growth conditions

All bacterial strains used in this study are listed in **Table S1**. *Mycobacterium smegmatis* mc^2^155 [10] and derived mutants [7], [8] were maintained on LB agar plates supplemented with 0.05% (w/v) Tyloxapol (Sigma-Aldrich). For broth culture, *M. smegmatis* was grown in Hartmans de Bont (HdB) minimal medium [11] supplemented with the stated carbon sources, 0.05% Tyloxapol, and 10 µM NiSO_4_. Cultures were incubated at 37°C with agitation (200 rpm) in 30 mL medium in 125 mL aerated conical flasks. Culture volumes were upscaled to 500 mL in 2.5 L flasks for transcriptome analysis and 100 mL in 500 mL flasks for metabolome analysis. Cells were inoculated to an initial optical density of 0.005. Optical densities to assess growth were measured at 600 nm (OD_600_) in a Jenway 6300 spectrometer. Cultures were diluted in 0.85% saline to bring the OD_600_ below 0.5 when measured in cuvettes of 1 cm light path length. To count colony forming units (CFU mL^−1^), each culture was serially diluted in phosphate-buffered saline (PBS) (pH 7.0) and spotted on to agar plates [12]. A markerless deletion of the Hyd2 large subunit (MSMEG_2719) was complemented with a pOLYG vector containing the *hyd2* operon (MSMEG_2720-2718) in order to minimise disruption to hydrogenase maturation and folding [8]. β-galactosidase assays and amperometric hydrogen measurements were performed as previously described [7].

### Challenge experiments

For acid challenge experiments, the strains were grown on HdB media at pH 7.0 to OD_600_ = 1.0. They were subsequently pelleted (7,000×g, 10 min, RT), washed in 100 mM citrate/phosphate buffer (pH 7.0), and resuspended in 100 mM citrate/phosphate buffer (pH 3.0 or pH 5.0). All buffer preparations contained 22 mM glycerol, 0.05% Tween80, and trace metals. Following acid challenge, the survival of cells was measured by measuring colony forming units (CFU mL^−1^). The minimum inhibitory concentrations (MICs) of pH 5.0-challenged cells to the protonophore carbonyl cyanide *m*-chlorophenylhydrazone (CCCP) was determined using serial dilutions as previously described [13].

### Measurement of internal pH and membrane potential

Internal pH and membrane potential was measured in *M. smegmatis* grown on HdB minimal medium at 2 h following the induction of stationary phase. Internal pH was calculated by determining the partitioning of a radioactive probe between intracellular and extracellular fractions. Cultures of 1 mL were incubated with 11 µM [^14^C] benzoate (10–25 mCi mmol^1^) (pH 7.5) (37°C, 10 min) and centrifuged through silicone oil (BDH Laboratory Supplies) (16,000×g, 5 min, RT). A 20 µl sample of the supernatant was removed. The tubes were otherwise frozen (−80°C, 60 min) and the cell pellets were removed with dog nail clippers. Samples of the supernatant (extracellular fraction) and pellet (intracellular fraction) were dissolved in scintillation fluid (Amersham). The relative concentrations of [^14^C] benzoate in each sample was measured using a LKB Wallac 1214 Rackbeta liquid scintillation counter (Perkin Elmer Life Sciences). The internal pH was calculated from the uptake of [^14^C] benzoate using the Henderson-Hasselbalch equation as previously described [14]. Membrane potential was measured by a equivalent method by detemining the partioning of 5 µM [^3^H] methyltriphenylphosponium iodide ([^3^H]TPP^+^) (30–60 Ci mmol^−1^). The membrane potential was calculated from the uptake of [^3^H]TPP^+^ using the Nernst equation [15].

### Measurement of [NAD^+^]/[NADH] ratios

1 mL cultures were centrifuged (150,000×g, 3 min, RT) and resuspended in either 0.3 mL 0.2 M HCl (for NAD^+^ extraction) or 0.3 mL 0.2 M NaOH (for NADH extraction). The cultures were heated (55°C, 10 min), cooled (0°C, 5 min), and neutralized with either 0.3 mL 0.1 M NaOH (for NAD^+^ extraction) or 0.3 mL 0.1 M HCl (for NADH extraction). After centrifugation (150,000×g, 3 min RT), the supernatants were collected. 200 µl supernatant was transferred into cuvettes containing 50 µl 1 M bicine (pH 8.0), 50 µl 40 mM EDTA (pH 8.0), 50 µl 4.2 mM 3-[4,5-dimethylthiazol-2-yl]-2,5-diphenyltetrazolium bromide, and 50 µl 16 mM phenazine ethosulfate. NAD^+^ and NADH concentrations were measured by addition of 50 µl ethanol and 5 U yeast alcohol dehydrogenase II [16]. The rate of reduction of 3-[4,5-dimethylthiazol-2-yl]-2,5-diphenyltetrazolium bromide was measured photometrically at 570 nm and was proportional to the concentration of standards of each cofactor.

### RNA extraction

*M. smegmatis* mc^2^155 and Δ*hyd*2 cells were grown synchronously in aerated conical flasks. At 1 hour following entry into stationary phase due to carbon-limitation, the cells were harvested for RNA extraction and microarray analysis. 500 mL of each culture were mixed with 1000 mL cold glycerol saline (3∶2 v/v) (-20°C), centrifuged (27000×g, 20 min, −20°C), and resuspended in glycerol saline (1∶1 v/v) (−20°C). Cell lysis was achieved by three cycles of bead-beating in a Mini-Beadbeater (Biospec) at 5,000 rpm for 30 sec. Total RNA was extracted using TRIzol reagent (Invitrogen) according to the manufacturer’s instructions. DNA was removed from the RNA preparation by treatment with 2 U RNase-free DNase using the TURBO DNA-*free* kit (Ambion), according to the manufacturer’s instructions. The concentration and purity of the RNA was determined using a NanoDrop ND-1000 spectrophotometer, and its integrity was confirmed on a 1.2% agarose gel.

### Microarray analysis

Transcriptome analysis employed glass slide DNA microarrays provided by the Pathogen Functional Genomics Research Center (PFGRC), which is funded by the National Institute of Allergy and Infectious Diseases. The arrays represented every open reading frame of the genome of *M. smegmatis* mc^2^155 with 7,736 unique 70-mers spotted in triplicate. Samples for microarray analysis were prepared and hybridised based on standard operating protocols (SOP) M007 and M008 from The Institute of Genomic Research (TIGR) [17]. 5 µg extracted total RNA was reverse-transcribed and aminoallyl (aa)-labelled using 3 µg random primers (Invitrogen), SuperScript III reverse trancriptase (Invitrogen), and a 25 mM aa-dUTP labelling mix (2∶3 aa-dUTP to dTTP) (Sigma-Aldrich). The synthesised cDNA was labelled with cyanine-3 (Cy3) or cyanine-5 (Cy5) fluorescent dyes (GE Healthcare BioSciences) for 2 h. After measurement of the concentration of cDNA and incorporated dyes (NanoDrop ND-1000 spectrophotometer), the labelled probes were mixed in equal ratios according to instructions in SOP M007. Prior to microarray hybridisation, the microarray slides were blocked, washed, and dried as described in SOP M008. The slides were immediately hybridised with the prepared samples and incubated overnight. After hybridisation, slides were washed with progressively more stringent buffers and dried as per SOP M008. Slides were immediately scanned using an Axon GenePix4000B microarray scanner (Molecular Devices) and analysed with the TM4 suite programs Spotfinder, MIDAS, and MeV as previously described [18]. Gene expression ratio (fold change from Δ*hyd*2 vs. wild-type) was calculated from the normalised signal intensities. Microarrays were hybridised using RNA from each of the four biological replicates. Cy3 and Cy5 dye swaps were employed between replicates.

### Quantitative RT-PCR

cDNA was synthesized from 1 µg of RNA for each sample with the SuperScript III Reverse Transcriptase Kit (Invitrogen). After cDNA synthesis, quantitative RT-PCR was performed using Platinum SYBR Green qPCR SuperMix-UDG with ROX (Invitrogen) according to the manufacturer’s instructions. Primers (Integrated DNA Technologies) for 10 genes (**Table S2**) were designed with the publicly available Primer3 software. Primer pairs were optimised to ensure efficient amplification. The real-time PCR reactions were conducted in ABI Prism 7500 (Applied Biosystems). Relative gene expression was determined from calculated threshold cycle (C*_T_*) values that were normalised to the gene *sigA* (MSMEG_2758) as an internal normalisation standard.

### Metabolome analysis

*M. smegmatis* mc^2^155 and Δ*hyd*2 cells were grown synchronously in aerated conical flasks. At 1 hour following entry into stationary phase due to carbon-limitation, samples were collected. To prepare samples for extracellular metabolite analysis, 15 mL of culture were centrifuged (27000×g, 10 min, RT) and the supernatant was stored at −20°C. To prepare samples for intracellular metabolite analysis, 15 mL of each culture were quenched with 30 mL cold glycerol saline (3∶2 v/v) (−20°C) [19], centrifuged (27000×g, 20 min, −20°C), and resuspended in 1 mL glycerol saline (1∶1 v/v) (−20°C) for storage. The samples were recentrifuged prior to metabolite extraction and the pellets were submitted to metabolite extraction. Before extraction, 20 µl of the internal standard (10 mM L-alanine-*d*_4_) was added to each intracellular and extracellular sample. The metabolites were extracted and derivatised as described in existing protocols [20]. The intracellular and extracellular metabolites were analysed using a gas chromatograph (GC-7890) coupled to a mass spectrometer (MSD5975) (Agilent Technologies) with a quadrupole mass selective detector (EI) operated at 70 eV. The results were processed using R package Metab [21], and samples were analysed using the mass spectral deconvolution and identification system [20]. The final concentration of metabolites was determined using the GC peak intensity of methyl chloroformate derivatives. Compounds considered false-positives were eliminated, and the intensity of each metabolite was normalised relative to the intensity of the internal standard. Samples of five technical replicates were collected and analysed from each of the three biological replicates.

## Results

### Hydrogen scavenging enhances survival of *M. smegmatis*

**Figure 1:**
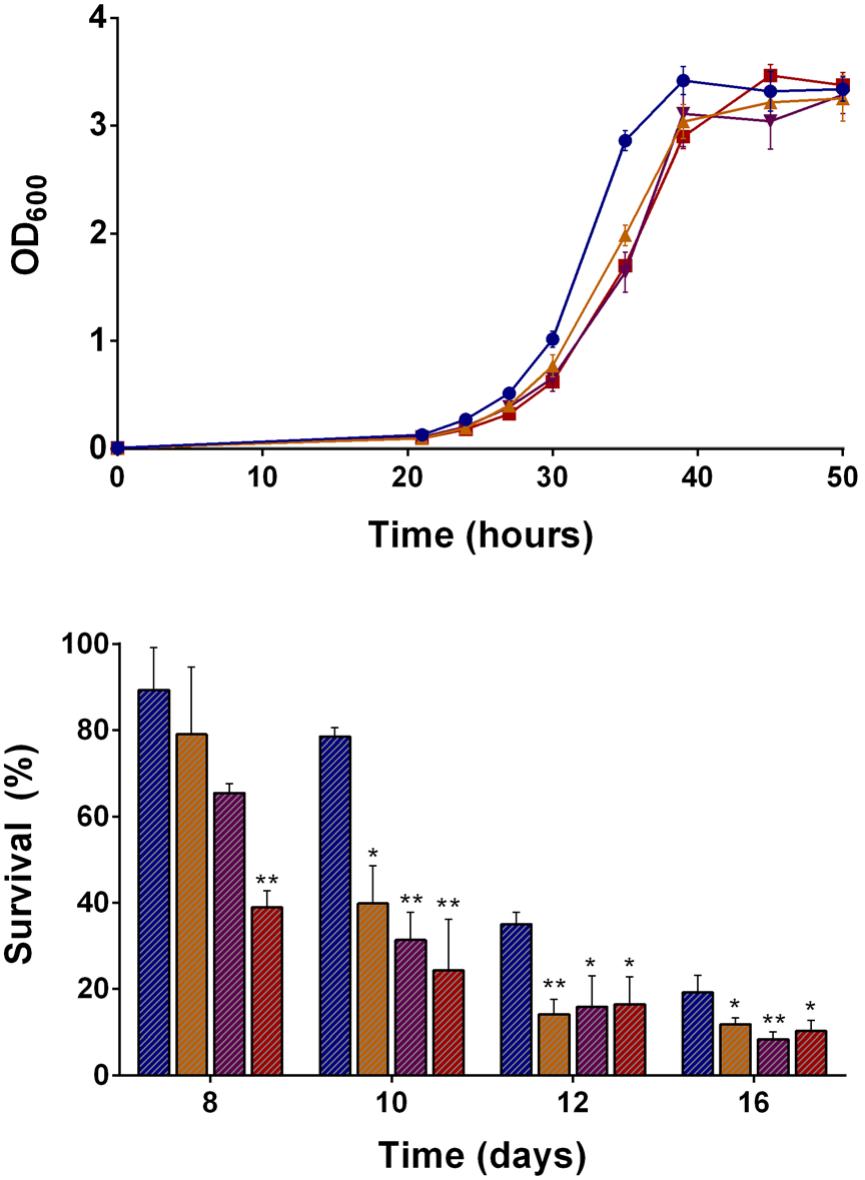
The growth and survival of *Mycobacterium smegmatis* in the presence and absences of hydrogenases. (A) Growth of *M. smegmatis* mc^2^155 and *hyd* mutants into carbon-limitation. (B) Survival of *M. smegmatis* mc^2^155 and *hyd* mutants during carbon-limitation. The strains were grown in aerated conical flasks on HdB minimal medium supplemented with 22 mM glycerol. Growth is shown in OD_600_. Survival is shown in percentage colony forming units relative to day four. Legend: Blue circles/bars = Wild-type; Red squares/bars = Δ*hyd*123; Orange point-up triangles/bars = Δ*hyd*1; and Purple point-down triangles/bars = Δ*hyd*2. Error bars show standard deviations from biological triplicates. * = *p*<0.05, ** = *p*<0.01, *** = *p*<0.001 difference relative to wild-type bars (Student’s T-test, unpaired, two-tailed).

To determine the physiological importance of H_2_ utilization, the growth and survival of *Mycobacterium smegmatis* mc^2^155 and hydrogenase mutants was measured in a range of conditions. All strains were grown in aerated conical flasks on HdB minimal salts medium supplemented with different carbon sources and the non-metabolisable detergent Tyloxapol. During growth on 22 mM glycerol (pH 7.0), there was no significant difference in the specific growth rates (∼0.25 h^−1^) or final growth yields (∼10^8^ CFU mL^−1^) of the strain lacking Hyd1 (Δ*hyd*1), Hyd2 (Δ*hyd*2), and all three hydrogenases (Δ*hyd*123) (**Figure 1A**). However, the long-term survival of these strains was compromised following the onset of carbon-limitation. Each strain lost viability at a significantly and reproducibly faster rate than the wild-type. At eight days and at all sampling points thereafter, the Δ*hyd*123 strain produced at least 40% fewer colony forming units compared to the wild-type. For example, after 12 days, 3.6×10^7^ CFU mL^−1^ were counted for the wild-type strain compared to 1.7×10^7^ CFU mL^−1^ for the Δ*hyd*123 strain The long-term survival of the Δ*hyd*1 and Δ*hyd*2 strains was also reduced (**Figure 1B**). Thus, atmospheric H_2_ is among the substrates that *M. smegmatis* employs to maintain viability when deprived of organic carbon sources.

### *M. smegmatis* grows optimally by co-metabolising organic carbon sources and atmospheric H_2_

**Figure 2:**
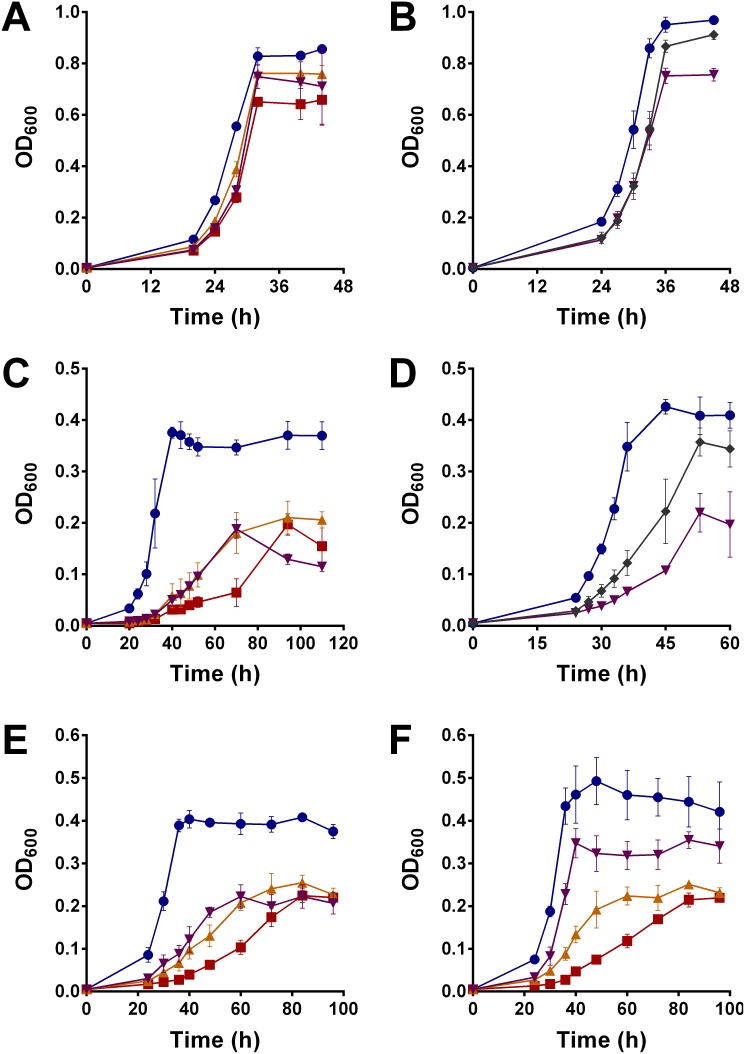
Observation, complementation, and recovery of mutant growth phenotypes. Strains were grown on HdB minimal medium. (A) Growth on 5.5 mM glycerol. (B) Complementation on 5.5 mM glycerol in the presence of 50 µg mL^−1^ hygromycin. (C) Growth on 12.5 mM acetate. (D) Partial complementation on 12.5 mM acetate in the presence of 50 µg mL^−1^ hygromycin. (E) Growth on 12.5 mM acetate in serum vials injected with 10% pure N_2_. (F) Growth on 12.5 mM acetate in serum vials injected with 10% pure H_2_. Legend: Blue circles = Wild-type (or wild-type with empty pOLYG vector for complementation); Red squares = Δ*hyd*123; Orange point-up triangles = Δ*hyd*1; Purple point-down triangles = Δ*hyd*2 (or Δ*hyd*2 with empty pOLYG vector for complementation); and Grey diamonds = Δ*hyd*2 with pOLYG vector expressing MSMEG_2720-2719. Error bars show standard deviations from biological triplicates.

Major growth phenotypes were observed when the hydrogenase mutants were grown at lower carbon concentrations or oxidised carbon sources. When the concentrations of glycerol was reduced from 22 mM to 5.5 mM, the Δ*hyd*123 strain grew to a final OD_600_ approximately 25% lower than the wild-type (**Figure 2A**). Growth of the mutant strain was also defective on a range of other carbon sources. There was a twofold reduction in the growth yield of the Δ*hyd*123 strain compared to the wild-type when the short-chain fatty acid acetate was available as the sole carbon source. Furthermore, the specific growth rate of the Δ*hyd*123 strain (0.03 h^−1^) was fivefold lower than the wild- type (0.17 h^−1^) (**Figure 2C**). When the phenotypes were traced to the single mutants, it was revealed that the growth of the Δ*hyd*1 and Δ*hyd*2 strains were intermediate to those of the wild-type and Δ*hyd*123 on 5.5 mM glycerol (**Figure 2A**) and 12.5 mM acetate (**Figure 2C**). These phenotypes indicate that these enzymes have overlapping roles during growth and survival, but are not redundant. It was possible to complement the Δ*hyd*2 phenotypes by expressing the genes of the operon MSMEG_2720-2718 from the Hsp60 promoter-driven hygromycin-resistant shuttle vector pOLYG [22] (**Figure 2B&D**); a reduction of growth rate was still observed in the complementation strain, likely due to inefficient hydrogenase maturation or differences in gene copy number, but the final growth yield was similar to the wild-type strain.

During growth on 12.5 mM acetate as the sole carbon source, it was also possible to partially recover the phenotype of the Δ*hyd*2 strain, but not the Δ*hyd*1 or Δ*hyd*123 strains, by growing the cells in the presence of 10% H_2_ (**Figure 2E&F**). The Δ*hyd*2 strain grew at a similar rate to the wild-type (µ_wt_ = 0.14 h^−1^; µ_Δ*hyd*2_ = 0.13 h^−1^; µ_Δ*hyd*1_ = 0.065 h^−1^) in this condition; these cells also attained a higher final growth yield (OD_600 wt_ = 0.45; OD_600 Δ*hyd*2_ = 0.35; OD_600 Δ*hyd*1_ = 0.22) on 10% H_2_ than when ambient H_2_ was absent (OD_600 wt_ = 0.40; OD_600 Δ*hyd*2_ = 0.21; OD_600 Δ*hyd*1_ = 0.22) (**Figure 2F**). No differences were observed when equivalent volumes of exogeneous N_2_ were introduced (**Figure 2E**). We model that Hyd1 (V_max (app)_ = 12 nmol g dw^−1 ^min^−1^ [7]) can compensate for the loss of Hyd2 by oxidising the majority of this exogenous H_2_. However, the converse compensation does not occur because the activity of the Hyd2 (V_max (app)_ = 2.5 nmol g dw^−1 ^min^−1^ [7]) strain is too low to consume exogenous H_2_ at rates meaningful for cell growth.

We tested whether the type of carbon source had an influence on hydrogenase expression or activity. Following growth on 5.5 mM glycerol compared to 12.5 mM acetate, the three hydrogenases were expressed at similar levels (**Figure S2A–C**) and whole-cells oxidised hydrogen at equivalent rates (**Figure S2D**). It was therefore clear that differences in hydrogenase activity were not accountable for the differences in the growth phenotypes. The Δ*hyd*2 strain grew to proportionately higher yields when stationary-phase cells were spiked with 12.5 mM acetate. During growth on 50 mM acetate, the Δ*hyd*2 strain also remained significantly impaired (µ = 0.08 h^−1^; OD_600 final_ = 1.2) compared to the wild-type (µ = 0.15 h^−1^; OD_600 final_ = 1.8). Repeated subculturing of acetate-grown cells also caused no improvement of growth rates or yields. Thus, the mutant cells consumed all the acetate provided in media, but less efficiently coupled its oxidation to growth.

### Δ*hyd*2 cells maintain redox balance, membrane potential, and pH gradients

It has been shown that both oxidative and evolving hydrogenases can be important for acid tolerance in *Enterobacteriaceae* [23], [24], [25]. We therefore tested the hypothesis that Hyd2 is required for intracellular pH homeostasis in *M. smegmatis*. Consistently, the hydrogenase deletions were significantly impaired compared to the wild-type when the external pH was lowered below pH 6.5 (**Figure S1A&B**); at pH 5.5, there was a significant difference in the specific growth rates (µ_wt_ = 0.12 h^−1^; µ_Δ*hyd*123_ = 0.05 h^−1^) and final growth yields (OD_600 wt_ = 0.24; OD_600 Δ*hyd*123_ = 0.11 h^−1^) of the strains (**Figure S1C**). However, the percentage survival, intracellular pH, and protonophore susceptibility of exponentially-grown cells challenged at pH 5.0 or pH 3.0 was similar between the strains (**Table S3**). It is therefore clear that the pH-dependent phenotypes do not reflect a defect in pH homeostasis and instead may be a secondary consequence of reduced electron input and altered metabolic flux in cells unable to scavenge H_2_.

Based on previous studies on the physiological roles of uptake hydrogenases [6], we predicted that the probable physiological role of Hyd2 was therefore to generate a proton-motive force through the respiratory chain and/or reduce coenzymes required for reductive processes. To distinguish these possibilities, we measured the membrane potential, pH gradient, and NAD^+^/NADH ratios of wild-type and Δ*hyd*2 strains following growth on 5.5 mM glycerol or 12.5 mM acetate. However, all three parameters were again similar between the wild-type and mutant strains and were within normal ranges (**Table 1**). We hypothesised that the energetic parameters of the Δ*hyd*2 strain were maintained at normal levels due to compensation of any deficiencies by organic electron acceptors.

**Table 1:** Energetic parameters of wild-type and Δ*hyd*2 cells two hours following the induction of stationary phase.

Carbon Source	5.5 mM Glycerol		12.5 mM Acetate	
Strain	WT	Δ*hyd*2	WT	Δ*hyd*2
**Growth Yield (OD_600_)**	0.86±0.01	0.73±0.04	0.37±0.03	0.19±0.03
**Growth Rate (h^−1^)**	0.19±0.04	0.17±0.01	0.17±0.01	0.07±0.01
**External pH**	6.3±0.1	6.5±0.1	7.5±0.1	7.5±0.1
**Internal pH**	7.3±0.2	7.1±0.1	7.1±0.1	7.2±0.2
**Membrane Potential (mV)**	−166±7	−163±5	−153±4	−155±9
**NAD^+^ Concentration (µM)**	1.6±0.4	1.6±0.1	0.90±0.14	0.95±0.12
**NADH Concentration (µM)**	3.6±0.5	2.6±0.5	0.55±0.14	0.53±0.12

Error margins represent standard deviations from three biological replicates.

### Organic electron donors compensate for loss of H_2_ oxidation

**Figure 3:**
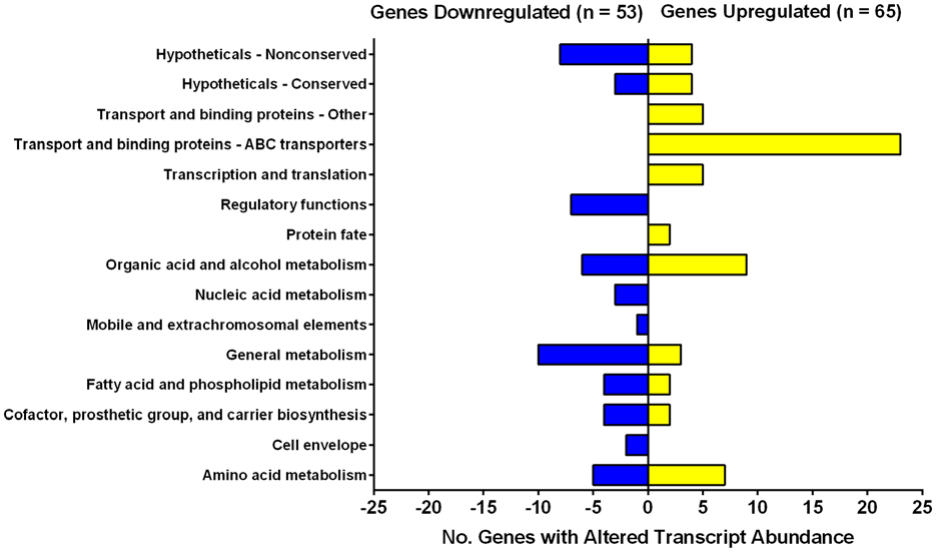
Genes with significant changes in expression in Δ*hyd*2 vs. wild-type cells. Both strains were grown synchronously on HdB minimal medium supplemented with 22>2.0, *p* value≤0.05. The genes were classified as significantly downregulated if expression ratio <0.5, *p* value≤0.05. The number of genes affected are listed by functional category. The full list of genes in each category is shown in **Table S4**, **Table S5**, and **Dataset S1**.

We performed a microarray analysis to confirm whether organic electron donors compensate for loss of tropospheric H_2_ oxidation. The transcriptome of Δ*hyd*2 versus wild-type cells was compared following growth on HdB minimal medium supplemented with 22 mM glycerol and 0.05% Tyloxapol. These conditions were selected because, while Hyd2 is expressed and active [7], [3], its deletion did not induce a significant growth phenotype aside from an extended lag phase (**Figure 1A**); cells therefore fully compensate for the loss of the expressed hydrogenases. In this condition, we determined that 65 genes were significantly upregulated (ratio >2.0; *p*≤0.05) (**Table S4**) and 53 genes were significantly downregulated (ratio <0.5; *p*≤0.05) (**Table S5**) in the Δ*hyd*2 strain compared to the wild-type (**Figure 3** **; Dataset S1**). The genes affected were distinct to those previously observed to be affected by slow growth or oxygen-limitation [8]. We performed quantitative RT-PCR to confirm the quality of the microarray data; the expression ratios of the selected genes correlated well with the microarray results (**Figure S3**).

The majority of the transcriptional changes involved genes implicated in substrate transport and binding, organic acid and alcohol metabolism, and amino acid metabolism (**Figure 3**). There were particularly extensive changes in central intermediary metabolism. MSMEG_3706, a bifunctional enzyme that catalyses key reactions in the glyoxylate shunt (isocitrate lyase) and methylcitrate cycle (methylcitrate lyase) [26], was significantly downregulated; this enzyme is usually upregulated during slow growth of *M. smegmatis* and *M. bovis BCG* [8]. In compensation, several predicted glycolytic and tricarboxylic acid cycle enzymes were upregulated, i.e. pyruvate dehydrogenase, isocitrate dehydrogenase, ketoglutarate-ferredoxin oxidoreductase [27], and lactate 2-monooxygenase [28]. All of these upregulated enzymes catalyse oxidative decarboxylation reactions that yield reduced cofactors concomitant with the loss of CO_2_. To compensate for downregulation of the methylisocitrate cycle [29], the strain also increased expression of the enzymes of the methylmalonyl-CoA pathway (propionyl-CoA carboxylase, methylmalonyl-CoA mutase) that converts propionate to succinate in an ATP-dependent manner [30]. These changes suggest that *M. smegmatis* compensates for loss of hydrogen oxidation by re-routing carbon flux from anabolic to catabolic pathways.

In amino acid metabolism, the operon encoding the determinants of the urea cycle was upregulated (MSMEG_3769-3773). These ATP-consuming enzymes convert the carbon components of amino acids into the tricarboxylic acid intermediate fumarate, while removing excess nitrogen as urea. Transcripts encoding the predicted NAD-dependent glutamate synthase MSMEG_6458-6459 were also significantly more abundant. We also observed that the putative operons encoding six ABC transporters were upregulated, including those predicted to transport trehalose, methionine, branched-chain amino acids, and alkane sulfonates. Some of these compounds may be scavenged from the cell envelope; it has previously been observed that trehalose is produced by mycobacteria as a byproduct of mycolic acid cell envelope biosynthesis, and the recycling of this compound by a homologous ABC transporter is essential for virulence in *Mycobacterium tuberculosis* [31].

We observed no significant changes in the expression of any hydrogenase-related genes in this condition, including Hyd1 (MSMEG_2263 ratio = 1.5) or Hyd3 (MSMEG_3928 ratio = 0.9), suggesting that other hydrogenases do not compensate for loss of Hyd2 in this condition.

### Δ*hyd*2 cells have an altered intracellular metabolome

**Figure 4:**
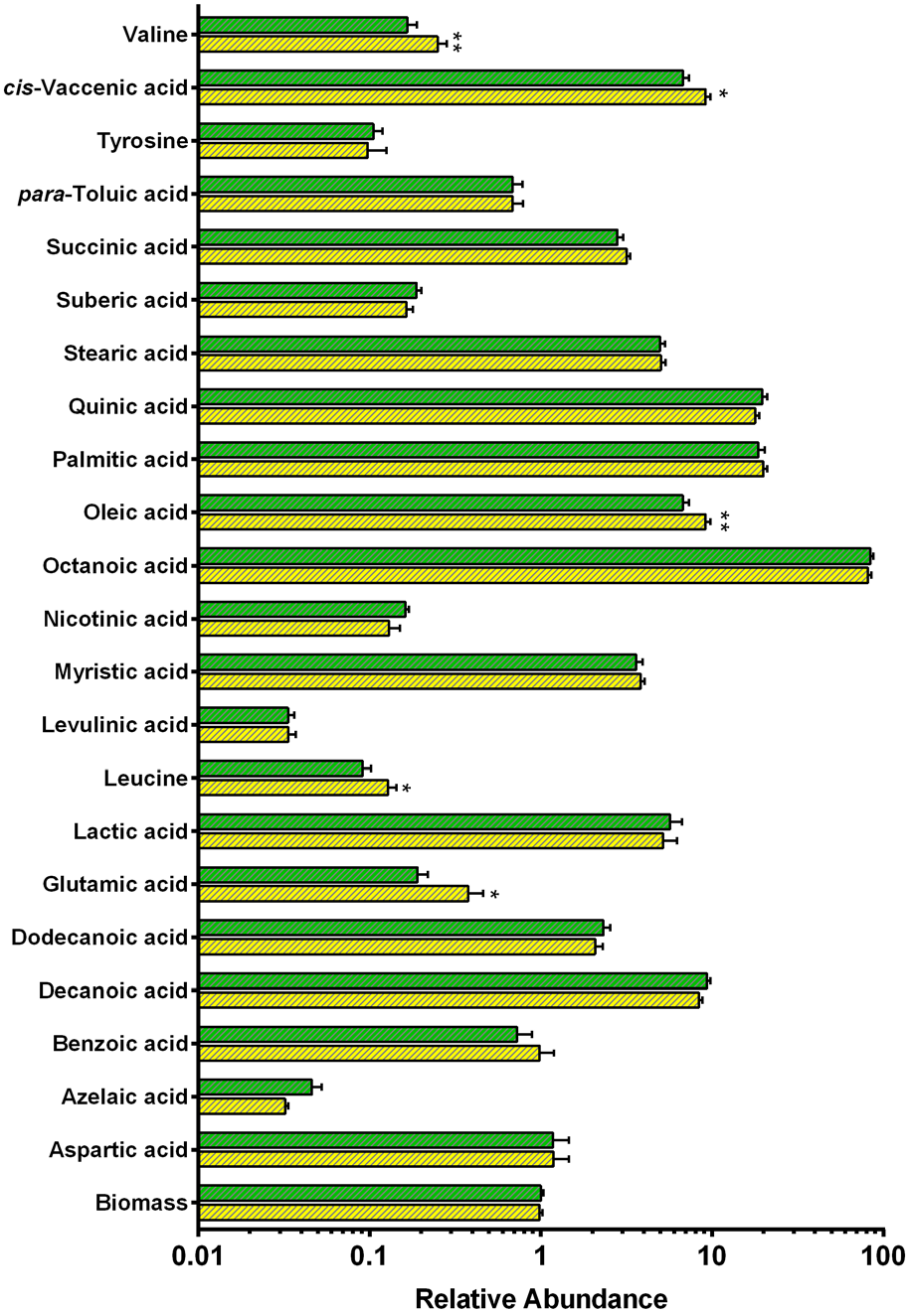
Profiles of intracellular metabolites in Δ*hyd*2 vs. wild-type cells. Metabolites were detected by gas chromatography-mass spectrometry (GC/MS). The values show the relative abundance of each metabolite detected in the samples (arbitrary units) on a logarithmic scale. Legend: Green = Wild-type, Yellow = Δ*hyd*2. Means were calculated from three biological replicates and five technical replicates for each strain. *p* values were determined using a Student’s T-test. * = *p*<0.05, ** = *p*<0.01 difference relative to wild-type bars.

The intracellular and extracellular metabolomes of Δ*hyd*2 and wild-type cells were determined by gas chromatography-mass spectrometry (GC/MS) under the same conditions as the microarray. The metabolome profile was generally similar between the strains, but there were statistically significant changes in the relative abundance of several amino acids and fatty acids (**Figure 4**; **Table S6**). These changes were consistent with the transcriptome data (**Table S4**; **Table S5**). The metabolome profile substantiates the finding that NAD-dependent glutamate synthase is upregulated in Δ*hyd*2 cells. There was a twofold increase in the concentration of glutamate in this strain. Consistent with the import or recycling of branched-chain amino acids, there was also 30–40% more leucine and valine inside Δ*hyd*2 cells (**Table S6**). This is in line with the mutant strains harnessing amino acids as electron donors in the absence of Hyd2.

## Discussion

In conclusion, it is clear that hydrogen scavenging enhances the growth and survival of *Mycobacterium smegmatis* under a range of conditions. Single and double markerless deletions of the hydrogen-scavenging enzymes Hyd1 or Hyd2 grew to lower yields than the wild-type strain. Mutant strains were defective when cultured on minimal medium at low carbon concentrations, acidic pH, and, most significantly, on short-chain fatty acids. Reduced growth yields of the Δ*hyd*2 strain have also been observed during growth on rich media, e.g. LBT (lysogeny broth supplemented with Tween80) [8]. All defects were observed when strains were grown in flasks aerated with ambient air, i.e. when H_2_ is available at trace concentrations. In high H_2_ atmospheres, the rapidly-oxidising hydrogenase Hyd1 could compensate for the loss of Hyd2, but the Δ*hyd*1 strain remained defective. *M. smegmatis* therefore grows optimally through mixotrophic metabolism of available carbon sources and a ubiqitious hydrogen supply. H_2_ scavenging is therefore likely to be a general feature of *M. smegmatis* growth and survival under physiological conditions, and may significantly influence the competitiveness of the bacterium in soil ecosystems.

**Figure 5:**
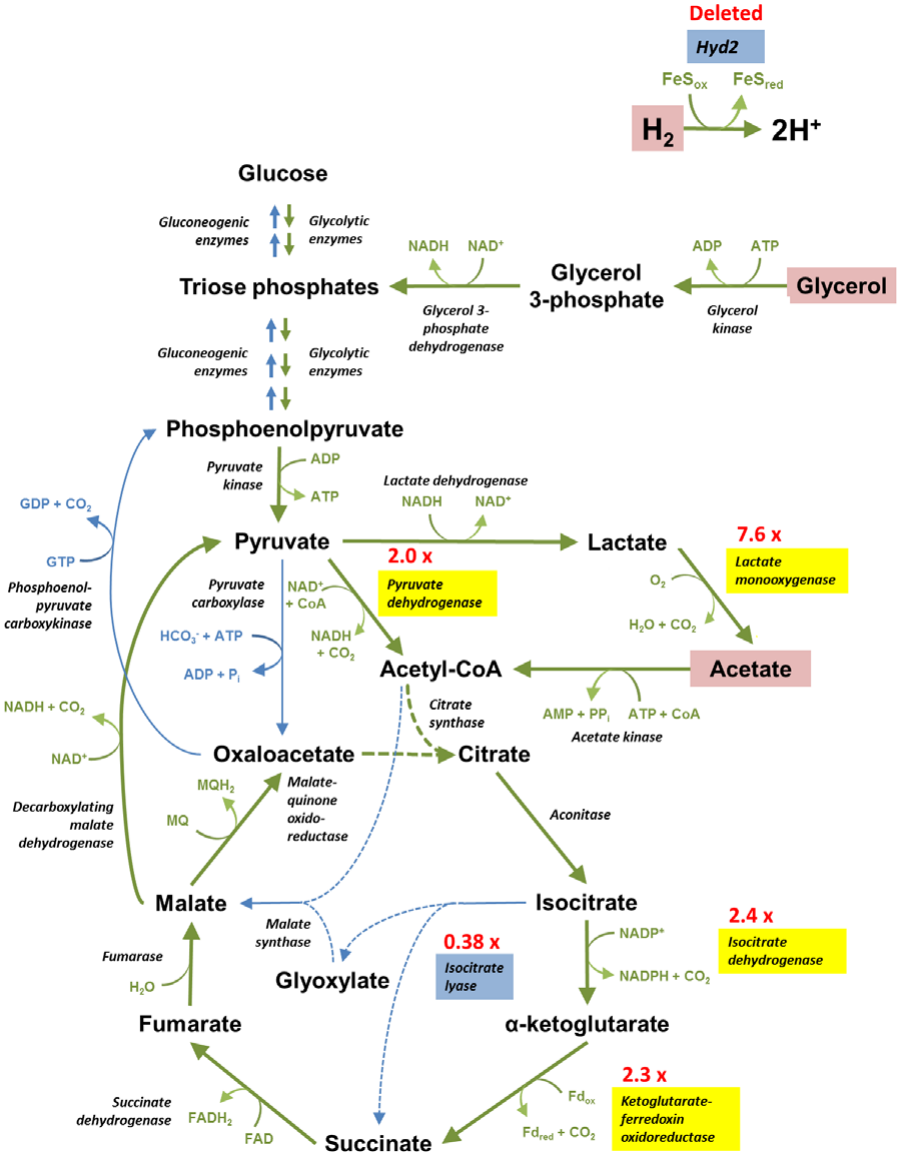
Altered balance of catabolic/anabolic carbon metabolism in Δ*hyd*2 cells. Cells lacking Hyd2 compensate for the loss of electrons derived from H_2_ by increasing oxidation of organic carbon sources. There is an increased flux though the tricarboxylic acid cycle due to upregulation of enzymes involved in oxidative decarboxylation (e.g. ketoglutarate-ferredoxin oxidoreductase) (highlighted in yellow) and downregulation of those involved in anaplerosis (i.e. isocitrate lyase) (highlighted in blue). We model that loss of CO_2_ through oxidative decarboxylation reactions is principally responsible for the decreased biomass of Δ*hyd*2 cells. Oxidative pathways are depicted with green arrows, whereas reductive pathways are represented with blue arrows. The red text shows the expression ratios of the significantly upregulated or downregulated genes in Δ*hyd*2 vs. wild-type cells.

Despite considerable investigation, it has proven challenging to resolve why hydrogen metabolism is so important during mixotrophic growth. The membrane potentials, pH gradients, and NAD^+^/NADH ratios of the mutant and wild-type strains were similar even in phenotype-inducing conditions. This is likely to be due to extensive compensation for the loss of hydrogen scavenging by organic carbon sources. Transcriptome and metabolome analysis inferred that the Δ*hyd*2 strain increased oxidation of organic carbon sources through central intermediary pathways. There was a pronounced increase in the transcript levels of the oxidative decarboxylation reactions of the tricarboxylic acid cycle, coupled with a downregulation of the glyoxylate shunt. Increased flux through oxidative pathways would increase the production of NADH, NADPH, and reduced ferredoxin with concomitant loss of CO_2_ (**Figure 5**). Mutant cells also upregulated several ABC importers for several potential organic electron donors, e.g. trehalose, methionine, and branched-chain amino acids, that may be scavenged from the cell envelope. In addition, metabolomics analysis showed that the amino acids glutamate, valine, and leucine were more abundant in the cells of the Δ*hyd*2 mutant. Transcriptomics indicate that they may be ultimately broken down into fumarate and urea through an upregulated urea cycle. Thus, amino acids may also serve as electron donors to compensate for loss of hydrogen scavenging.

To reconcile the available evidence, we propose that tropospheric H_2_ principally serves as a source of reductant in carbon metabolism during exponential growth of *M. smegmatis*. The electrons yielded from its oxidation may provide the reduced compounds required for efficient carbon metabolism. This process enables cells to efficiently balance anabolic and catabolic processes to maximise yields during mixotrophic growth. Reductant is likely to be especially important during growth on acetate. This compound is more oxidised than glycerol and its metabolism results in higher NAD^+^/NADH ratios (**Table 1**). In addition, acetate molecules cannot be simultaneously used for catabolic and anabolic reactions due to loss of CO_2_. Increased flux through catabolic reactions in the Δ*hyd*2 strain on acetate would therefore cause reduced biomass via loss of carbon as CO_2_. Membrane-association may be an advantage in this case because it enables the hydrogenases to more efficiently bind the extracellular H_2_ that diffuses into cells.

We have previously postulated that Hyd1 and Hyd2 enzymes directly couple the oxidation of H_2_ to the reduction of O_2_ via the electron transport chain [7]. The membrane association and oxygen-dependence of hydrogenase activity indicates it is physically and functionally linked to the respiratory chain [3], [7]. However, it seems improbable that oxidation of nanomolar concentrations of H_2_ could significantly influence proton-motive generation during exponential growth on millimolar concentrations of carbon sources; our phenotypic and transcriptome studies are more consistent with hydrogenases harnessing electrons for reductive cellular processes. It nevertheless remains conceivable that aerobic hydrogen respiration may be responsible for the enhanced long-term survival of wild-type cells compared to Δ*hyd*123 cells during carbon-limitation. Hydrogenases are expressed at higher levels [8] and oxidise tropospheric H_2_ more rapidly [7] in this condition. Tropospheric H_2_ oxidation may therefore serve as a significant generator of proton-motive force when organic carbon supplies are exhausted; H_2_ is a dependable fuel source given it is present at a constant, albeit trace, concentration throughout the troposphere. Expression and activity profiling suggests that Group 5 [NiFe]-hydrogenases have equivalent roles during the sporulation of streptomycetes and the adaptation of rhodococci to carbon-limitation [2], [4].

The processes of using hydrogenases to generate reductant and generate proton-motive force need not be mutually exclusive. The NADH generated by the Group 3d [NiFe]-hydrogenase of *R. eutropha*, for example, can be simultaneously oxidised in the respiratory chain and used as reductant in the Calvin cycle [6], [32], [33]. Tropospheric H_2_ oxidation may also be coupled to the reduction of a multifunctional redox carrier in *M. smegmatis*. Identification and characterisation of the electron acceptors of Hyd1 and Hyd2 is clearly a priority in order to elucidate the cellular processes where these enzymes contribute.

## Supporting Information

Figure S1 **Importance of hydrogen metabolism for growth at acidic pH.** All strains were grown on HdB medium supplemented with 22 mM glycerol. pH was adjusted with concentrated HCl. (A) Final growth yield of strains grown at a range of pHs. (B) Specific growth rate of strains grown at a range of pHs. (C) Full growth curve of strains at pH 5.5. Legend: Blue circles = wild-type, Red squares = Δ*hyd*123; Orange point-up triangles = Δ*hyd*1; Purple point-down triangles = Δ*hyd*2. Error bars show standard deviations from biological triplicates.
(TIF)

Figure S2 **Expression and activity of hydrogenases on glycerol and acetate.** (A) *hyd1*, (B) *hyd2*, and (C) *hyd3* expression measured with promoter-*lacZ* reporter plasmids. *M. smegmatis* mc^2^155 harbouring either pJEM*hyd1-lacZ*, pJEM*hyd2-lacZ*, or pJEM*hyd3-lacZ* were grown in HdB minimal medium supplemented with either 5.5 mM glycerol or 12.5 mM acetate. Samples for β-galactosidase activity assays were withdrawn in mid-exponential (yellow bars) and early stationary (green bars) phase. (D) Hydrogenase activity of whole-cells grown in HdB minimal medium supplemented with 5.5 mM glycerol or 12.5 mM acetate during early stationary phase. Activity was measured amperometrically. Positive values indicate net H_2_ evolution. Negative values indicate net H_2_ consumption. Error bars show standard deviations from three biological replicates.
(TIF)

Figure S3 **Validation of microarray data by quantitative RT-PCR.** Microarray data were validated by comparing the gene expression changes of selected genes with that of qRT-PCR using the same RNA samples. Genes were chosen that were downregulated (MSMEG_1203, MSMEG_3706), upregulated (MSMEG_3194, MSMEG_3249, MSMEG_3962, MSMEG_3769, MSMEG_5059), or unchanged (MSMEG_4640) in the microarray. All bars show the expression ratio of genes in Δ*hyd*2 vs. wild-type strains. Yellow bars show microarray data. Green bars show qRT-PCR data. Error bars represent standard deviations from four biological replicates. * = *p*<0.05, ** = *p*<0.01 difference relative to zero (Student’s T-test).
(TIF)

Table S1 **Bacterial strains and plasmids used in this work.**
(DOCX)

Table S2 **qRT-PCR primers used in this study.** The forward and reverse primers for each gene (MSMEG_XXXX) targeted is listed.
(DOCX)

Table S3 **Intracellular pH homeostasis of** ***M. smegmatis*** **mc^2^155 following acid challenge.** Percentage survival, internal pH, and protonophore susceptibility of wild-type and *hyd* mutants is shown following acid exposure. Cultures were grown on HdB supplemented with 22 mM glycerol to OD 1.0. Cells were subsequently challenged in 100 mM citrate/phosphate buffer at pH 5.0 or pH 3.0. Error margins show standard deviations from three biologically independent replicates.
(DOCX)

Table S4 **Genes significantly upregulated in Δ** ***hyd*** **2 vs. wild-type microarrays.** Means and *p* values are calculated from four microarrays. The genes were classified as significantly upregulated if expression ratio >2.0, *p* value≤0.05. Less stringent criteria was sometimes used when genes were operonic with other upregulated genes, or when *p* values were perturbed by one clearly anomalous replicate. Asterisks are placed next to genes that did not meet the strict criteria, but are still very likely to be upregulated in the Δ*hyd*2 strain.
(DOCX)

Table S5 **Genes significantly downregulated in Δ** ***hyd*** **2 vs. wild-type microarray.** The mean gene expression ratio was calculated from the normalised signal intensities of four microarrays. The genes were classified as significantly downregulated if expression ratio <0.5 and *p* value≤0.05 (Student’s T test). Less stringent criteria was sometimes used when genes were operonic with other downregulated genes, or when *p* values were perturbed by one clearly anomalous replicate. Asterisks are placed next to such genes that did not meet the strict criteria, but are still very likely to be downregulated in the Δ*hyd*2 strain.
(DOCX)

Table S6 **List of intracellular and extracellular metabolites in Δ** ***hyd*** **2 vs. wild-type cells.** Metabolites were detected by gas chromatography-mass spectrometry (GC/MS). Values show the relative abundance of each metabolite detected in the samples (arbitrary units). Means were calculated from three biological replicates and five technical replicates for each strain and are shown to two significant figures. *p* values were determined using a Student’s T-test. Changes were classified as significant when *p*≤0.05 (Student’s T-test).
(DOCX)

Dataset S1 **All genes with significant changes in expression ratios comparing Δ** ***hyd*** **2 to WT.** See excel file.
(XLSX)
