# Supplementary material for: The Growth and Survival of Mycobacterium smegmatis Is Enhanced by Co-Metabolism of Atmospheric H2
Source: PLoS One. 2014 Jul 24;9(7):e103034. doi: 10.1371/journal.pone.0103034 (PMC4109961; doi:10.1371/journal.pone.0103034)
Supplement: Table S1 — Bacterial strains and plasmids used in this work. (DOCX) [file pone.0103034.s004.docx]

| **Strain** | **Description** | **Reference** |
| --- | --- | --- |
|  |  |  |
| ***Mycobacterium smegmatis*** | |  |
| mc^2^155 | Electrocompetent wild-type strain of *M. smegmatis* | **10** |
| ∆*hyd*1 | mc^2^155 with markerless deletion of MSMEG_2262 | **7** |
| ∆*hyd*2 | mc^2^155 with markerless deletion of MSMEG_2719 | **8** |
| ∆*hyd*3 | mc^2^155 with markerless deletion of MSMEG_3931 | **7** |
| ∆*hyd*123 | mc^2^155 with markerless deletions of MSMEG_2262, MSMEG_2719, MSMEG_3931 | **7** |
| **Plasmids** |  |  |
| pOLYG | *Escherichia* *coli*-mycobacterial shuttle vector with Hsp60 promoter; Hyg^r^ | **22** |
| pOLYGHyd2 | pOLYG carrying MSMEG_2720-2718 for complementation | **8** |
| pJEM*hyd1*-*lacZ* | pJEM15 containing *lacZ* gene fused to the promoter of the *hyd1* operon | **7** |
| pJEM*hyd2*-*lacZ* | pJEM15 containing *lacZ* gene fused to the promoter of the *hyd2* operon | **7** |
| pJEM*hyd3-lacZ* | pJEM15 containing *lacZ* gene fused to the promoter of the *hyd3* operon | **7** |
