# Supplementary material for: The Growth and Survival of Mycobacterium smegmatis Is Enhanced by Co-Metabolism of Atmospheric H2
Source: PLoS One. 2014 Jul 24;9(7):e103034. doi: 10.1371/journal.pone.0103034 (PMC4109961; doi:10.1371/journal.pone.0103034)
Supplement: Table S2 — qRT-PCR primers used in this study. The forward and reverse primers for each gene (MSMEG_XXXX) targeted is listed. (DOCX) [file pone.0103034.s005.docx]

| **Name** | **Sequence (5’-3’)** |
| --- | --- |
|  |  |
| 1203FW | GTTCGGGAAGTCGAGGTACA |
| 1203RV | CTTGTAGATGAACCGCGTGA |
| 3194FW | CAACGGCACAGATGTACTGG |
| 3194RV | GGTCTTGAGGCTGATGATGC |
| 3249FW | CTCGATGCTGTTCCTGTGTG |
| 3249RV | GCGAGTCCGAAGAACAGGTA |
| 3706FW | GCCCAGAAGAAGAGCATCAC |
| 3706RV | GAACCTGACTCAGCGGGTAG |
| 3769FW | GTTACGTCACGTTGCACGAC |
| 3769RV | TTCCTTGTCCTCCTGCAGAT |
| 3962FW | CACTTCCACTCGAGGAGAGC |
| 3962RV | GGCCACGTAGGACAACACTC |
| 5059FW | CTGGCGATCTACACGCTCTC |
| 5059RV | GCGAAGATGAACACCAGGAT |
| 6459FW | GTTCTCCACCAACACGTTCC |
| 6459RV | ACACCGGCACTATCTTGTCC |
| SigAFW | GACTCTTCCTCGTCCCACAC |
| SigARV | GAAGACACCGACCTGGAACT |
|  |  |
