# Supplementary material for: The Growth and Survival of Mycobacterium smegmatis Is Enhanced by Co-Metabolism of Atmospheric H2
Source: PLoS One. 2014 Jul 24;9(7):e103034. doi: 10.1371/journal.pone.0103034 (PMC4109961; doi:10.1371/journal.pone.0103034)
Supplement: Table S3 — Intracellular pH homeostasis of M. smegmatis mc2155 following acid challenge. Percentage survival, internal pH, and protonophore susceptibility of wild-type and hyd mutants is shown following acid exposure. Cultures were grown on HdB supplemented with 22 mM glycerol to OD 1.0. Cells were subsequently challenged in 100 mM citrate/phosphate buffer at pH 5.0 or pH 3.0. Error margins show standard deviations from three biologically independent replicates. (DOCX) [file pone.0103034.s006.docx]

|  | **WT** | **Δ*hyd*123** | **Δ*hyd*1** | **Δ*hyd*2** |
| --- | --- | --- | --- | --- |
|  |  |  |  |  |
| Survival (pH 5.0, 8 h) (%) | 74 ± 19 | 75 ± 20 | 95 ± 4 | 88 ± 8 |
| Survival (pH 3.0, 8 h) (%) | 0.8 ± 0.3 | 1.1 ± 0.3 | 0.9 ± 0.5 | 1.1 ± 0.4 |
| Internal pH (pH 3.0, 4 h) | 6.3 ± 0.2 | 6.7 ± 0.2 | 6.8 ± 0.1 | 6.8 ± 0.3 |
| MIC for CCCP (pH 5.0) (µM) | 10 | 10 | 10 | 10 |
