# Supplementary material for: The Growth and Survival of Mycobacterium smegmatis Is Enhanced by Co-Metabolism of Atmospheric H2
Source: PLoS One. 2014 Jul 24;9(7):e103034. doi: 10.1371/journal.pone.0103034 (PMC4109961; doi:10.1371/journal.pone.0103034)
Supplement: Table S4 — Genes significantly upregulated in Δ hyd 2 vs. wild-type microarrays. Means and p values are calculated from four microarrays. The genes were classified as significantly upregulated if expression ratio >2.0, p value≤0.05. Less stringent criteria was sometimes used when genes were operonic with other upregulated genes, or when p values were perturbed by one clearly anomalous replicate. Asterisks are placed next to genes that did not meet the strict criteria, but are still very likely to be upregulated in the Δhyd2 strain. (DOCX) [file pone.0103034.s007.docx]

|  | |  |  |  |  |
| --- | --- | --- | --- | --- | --- |
| **Locus** | | **Predicted function** | **Mean** | ***p*** |  |
|  | |  |  |  |  |
| **Amino acid biosynthesis** | | |  |  | |
| MSMEG_1652 | O-acetylhomoserine aminocarboxypropyltransferase | | 3.58 | 0.047 | |
| MSMEG_3769 | Argininosuccinate lyase | | 3.04 | 0.033 | |
| MSMEG_3770 | Argininosuccinate synthase | | 2.00 | 0.014 | |
| MSMEG_3772 | Ornithine carbamoyltransferase | | 2.58 | 0.037 | |
| MSMEG_3773 | Acetylornithine aminotransferase | | 2.17 | 0.029 | |
| MSMEG_6458 | Glutamate synthase (NAD-dependent), small subunit | | 2.97 | 0.032 | |
| MSMEG_6459 | Glutamate synthase (NAD-dependent), large subunit | | 2.89 | 0.046 | |
|  |  | |  |  | |
| **Cofactor, prosthetic group, and carrier biosynthesis** | | | |  | |
| MSMEG_3194 | Biotin synthase | | 2.71 | 0.010 | |
| MSMEG_4976 | Isochorismatase | | 2.19 | 0.029 | |
|  |  | |  |  | |
| **Fatty acid and phospholipid metabolism** | | |  |  | |
| MSMEG_0760 | Thioesterase family protein | | 2.29 | 0.025 | |
| MSMEG_5941 | 3-ketosteroid dehydrogenase | | 2.03 | 0.032 | |
|  |  | |  |  | |
| **General metabolism** | | |  |  | |
| MSMEG_6267 | Thiocyanate hydrolase, gamma subunit | | 2.17 | 0.004 | |
| MSMEG_6384 | Catalase / peroxidase HPI | | 3.03 | 0.019 | |
| MSMEG_6752 | Endoglucanase | | 2.76 | 0.047 | |
|  |  | |  |  | |
| **Organic acid and alcohol metabolism** | | |  |  | |
| MSMEG_1654 | Isocitrate dehydrogenase (NADP-dependent)* | | 2.44 | 0.201 | |
| MSMEG_3158 | Methylmalonyl-CoA mutase, small subunit | | 2.80 | 0.032 | |
| MSMEG_3159 | Methylmalonyl-CoA mutase large subunit | | 2.43 | 0.014 | |
| MSMEG_3962 | Lactate 2-monooxygenase* | | 7.64 | 0.166 | |
| MSMEG_3964 | Pyruvate dehydrogenase | | 2.02 | 0.020 | |
| MSMEG_4645 | Ketoglutarate-ferredoxin oxidoreductase, beta subunit* | | 1.76 | 0.084 | |
| MSMEG_4646 | Ketoglutarate-ferredoxin oxidoreductase, alpha subunit* | | 2.32 | 0.066 | |
| MSMEG_5939 | Acetaldehyde dehydrogenase | | 2.12 | 0.027 | |
| MSMEG_6391 | Propionyl-CoA carboxylase, beta chain | | 2.41 | 0.049 | |
|  |  | |  |  | |
| **Protein fate** | | |  |  | |
| MSMEG_2092 | D-aminopeptidase | | 2.21 | 0.046 | |
| MSMEG_3034 | Metallopeptidase family protein | | 2.03 | 0.042 | |
|  |  | |  |  | |
| **Transcription and translation** | | |  |  | |
| MSMEG_1442 | Ribosomal protein S3 | | 3.28 | 0.046 | |
| MSMEG_3778 | Phenylalanyl-tRNA synthetase, alpha subunit | | 2.19 | 0.012 | |
| MSMEG_4674 | Trigger factor | | 2.72 | 0.048 | |
| MSMEG_5431 | Ribosomal protein L25, Ctc-form | | 2.89 | 0.039 | |
| MSMEG_6413 | Seryl-tRNA synthetase | | 2.86 | 0.015 | |
|  |  | |  |  | |
| **Transport and binding proteins – ABC transporters** | | |  |  | |
| MSMEG_0549 | Alkane sulfonate ABC transporter, permease subunit* | | 5.13 | 0.070 | |
| MSMEG_0550 | Alkane sulfonate ABC transporter, binding protein* | | 5.32 | 0.055 | |
| MSMEG_0551 | Alkane sulfonate ABC transporter, ATPase subunit | | 3.42 | 0.049 | |
| MSMEG_3056 | Methionine ABC transporter, ATPase subunit* | | 1.81 | 0.054 | |
| MSMEG_3057 | Methionine ABC transporter, permease subunit* | | 2.46 | 0.127 | |
| MSMEG_3058 | Methionine ABC transporter, binding protein* | | 2.28 | 0.141 | |
| MSMEG_3247 | Branched-chain amino acid ABC transporter, binding protein* | | 2.62 | 0.169 | |
| MSMEG_3248 | Branched-chain amino acid ABC transporter, permease subunit | | 2.23 | 0.046 | |
| MSMEG_3249 | Branched-chain amino acid ABC transporter, permease subunit | | 2.98 | 0.033 | |
| MSMEG_3250 | Branched-chain amino acid ABC transporter, ATPase subunit* | | 2.49 | 0.065 | |
| MSMEG_3251 | Branched-chain amino acid ABC transporter, ATPase subunit* | | 2.24 | 0.256 | |
| MSMEG_4530 | Sulfate ABC transporter, ATP-binding protein* | | 3.88 | 0.081 | |
| MSMEG_4531 | Sulfate ABC transporter, permease subunit | | 5.42 | 0.004 | |
| MSMEG_4532 | Sulfate ABC transporter, permease subunit* | | 4.69 | 0.099 | |
| MSMEG_4533 | Sulfate ABC transporter, binding protein* | | 5.99 | 0.137 | |
| MSMEG_4557 | Cobalamin / Fe^3+^ siderophore ABC transporter, ATPase subunit* | | 3.81 | 0.261 | |
| MSMEG_4559 | Cobalamin / Fe^3+^ siderophore ABC transporter, permease subunit* | | 2.31 | 0.069 | |
| MSMEG_4560 | Cobalamin / Fe^3+^ siderophore ABC transporter, binding protein* | | 2.58 | 0.088 | |
| MSMEG_4561 | Cobalamin / Fe^3+^ siderophore ABC transporter, binding protein | | 3.08 | 0.045 | |
| MSMEG_5058 | Trehalose ABC transporter, ATPase subunit | | 2.53 | 0.025 | |
| MSMEG_5059 | Trehalose ABC transporter, permease subunit | | 2.98 | 0.034 | |
| MSMEG_5060 | Trehalose ABC transporter, permease subunit | | 2.07 | 0.043 | |
| MSMEG_5061 | Trehalose ABC transporter, binding protein | | 2.08 | 0.046 | |
|  |  | |  |  | |
| **Transport and binding proteins – other** | | |  |  | |
| MSMEG_2303 | Integral membrane transporter | | 3.33 | 0.025 | |
| MSMEG_2304 | Putative transporter | | 2.35 | 0.026 | |
| MSMEG_2332 | Amino acid carrier protein | | 3.14 | 0.017 | |
| MSMEG_2619 | Efflux protein | | 2.48 | 0.048 | |
| MSMEG_3045 | Integral membrane protein | | 2.87 | 0.040 | |
|  |  | |  |  | |
| **Hypotheticals – conserved** | | |  |  | |
| MSMEG_0077 | Conserved hypothetical protein | | 2.30 | 0.003 | |
| MSMEG_0141 | Transmembrane protein | | 2.00 | 0.046 | |
| MSMEG_0841 | Conserved hypothetical protein, frameshifted | | 2.03 | 0.022 | |
| MSMEG_6649 | Conserved hypothetical protein | | 2.57 | 0.029 | |
|  |  | |  |  | |
| **Hypotheticals – nonconserved** | | |  |  | |
| MSMEG_1394 | Transmembrane protein | | 2.08 | 0.049 | |
| MSMEG_3966 | Hypothetical protein | | 3.86 | 0.027 | |
| MSMEG_5325 | Hypothetical protein | | 2.14 | 0.034 | |
|  |  | |  |  | |
|  |  | |  |  | |
