# Supplementary material for: The Growth and Survival of Mycobacterium smegmatis Is Enhanced by Co-Metabolism of Atmospheric H2
Source: PLoS One. 2014 Jul 24;9(7):e103034. doi: 10.1371/journal.pone.0103034 (PMC4109961; doi:10.1371/journal.pone.0103034)
Supplement: Table S5 — Genes significantly downregulated in Δ hyd 2 vs. wild-type microarray. The mean gene expression ratio was calculated from the normalised signal intensities of four microarrays. The genes were classified as significantly downregulated if expression ratio <0.5 and p value≤0.05 (Student’s T test). Less stringent criteria was sometimes used when genes were operonic with other downregulated genes, or when p values were perturbed by one clearly anomalous replicate. Asterisks are placed next to such genes that did not meet the strict criteria, but are still very likely to be downregulated in the Δhyd2 strain. (DOCX) [file pone.0103034.s008.docx]

|  |  | | |  |  | |
| --- | --- | --- | --- | --- | --- | --- |
| **Locus** | **Predicted function** | | | **Mean** | ***p*** | |
|  |  | | |  |  | |
| **Amino acid metabolism** | | | |  |  | |
| MSMEG_1488 | Acetolactate synthase* | | | 0.29 | 0.069 | |
| MSMEG_1491 | Histidinol-phosphate aminotransferase 2 | | | 0.24 | 0.043 | |
| MSMEG_3326 | Carboxylate-amine ligase | | | 0.48 | 0.033 | |
| MSMEG_3442 | Cyclohexadienyl dehydratase | | | 0.46 | 0.019 | |
| MSMEG_5389 | Diaminopimelate decarboxylase (LysA) | | | 0.37 | 0.047 | |
|  |  | | |  |  | |
| **Cell envelope** | | | |  |  | |
| MSMEG_1203 | Methoxy-mycolic acid synthase | | | 0.40 | 0.046 | |
| MSMEG_4932 | UDP-N-acetylglucosamine carboxyvinyltransferase | | | 0.48 | 0.001 | |
|  |  | | |  |  | |
| **Cofactor, prosthetic group, and carrier biosynthesis** | |  | | | | |
| MSMEG_2809 | Polyketide cyclase | | | 0.48 | 0.009 | |
| MSMEG_3667 | Para-nitrobenzyl esterase | | | 0.48 | 0.029 | |
| MSMEG_3797 | Esterase family protein | | | 0.36 | 0.030 | |
| MSMEG_5364 | Amidohydrolase 2 | | | 0.29 | 0.048 | |
|  |  | | |  |  | |
| **Fatty acid and phospholipid metabolism** | | | |  |  | |
| MSMEG_1490 | 3-Oxoacyl-ACP synthase III* | | | 0.25 | 0.052 | |
| MSMEG_3321 | Carnitine transferase | | | 0.49 | 0.048 | |
| MSMEG_5739 | Long-chain fatty-acid CoA ligase | | | 0.25 | 0.044 | |
| MSMEG_2161 | Fatty acid CoA-ligase, frameshifted | | | 0.15 | 0.008 | |
|  |  | | |  |  | |
| **General metabolism** | | | |  |  | |
| MSMEG_0811 | Monooxygenase | | | 0.27 | 0.045 | |
| MSMEG_1475 | Monooxygenase | | | 0.29 | 0.010 | |
| MSMEG_1479 | Radical SAM methyltransferase | | | 0.38 | 0.023 | |
| MSMEG_1480 | Radical SAM methyltransferase | | | 0.36 | 0.033 | |
| MSMEG_1996 | F_420_-dependent oxidoreductase | | | 0.45 | 0.019 | |
| MSMEG_2310 | Monooxygenase | | | 0.46 | 0.030 | |
| MSMEG_4973 | Glyoxalase / dioxygenase | | | 0.47 | 0.048 | |
| MSMEG_4986 | Glycosyltransferase | | | 0.37 | 0.004 | |
| MSMEG_4987 | Glycosyltransferase | | | 0.37 | 0.003 | |
| MSMEG_5334 | Oxidoreductase | | | 0.32 | 0.046 | |
|  |  | | |  |  | |
| **Mobile and extrachromosomal elements** | | | |  |  | |
| MSMEG_2747 | Transposase | | | 0.45 | 0.011 | |
|  |  | | |  |  | |
| **Nucleic acid metabolism** | | | |  |  | |
| MSMEG_1291 | Xanthine dehydrogenase | | | 0.48 | 0.041 | |
| MSMEG_1292 | Xanthine dehydrogenase* | | | 0.53 | 0.070 | |
| MSMEG_5328 | dUTPase | | | 0.46 | 0.041 | |
|  |  | | |  |  | |
| **Organic acid and alcohol metabolism** | | | |  |  | |
| MSMEG_2121 | Multiphosphoryl transfer protein | | | 0.46 | 0.020 | |
| MSMEG_2122 | Dihydroxyacetone kinase, L subunit | | | 0.49 | 0.040 | |
| MSMEG_2123 | Dihydroxyacetone kinase, K subunit | | | 0.48 | 0.033 | |
| MSMEG_2254 | Oxalate decarboxylase | | | 0.42 | 0.026 | |
| MSMEG_3706 | Isocitrate lyase | | | 0.38 | 0.038 | |
| MSMEG_5164 | Zinc-binding alcohol dehydrogenase | | | 0.39 | 0.041 | |
|  |  | | |  |  | |
| **Regulatory functions** | | | |  |  | |
| MSMEG_0742 | LysR family transcriptional regulator | | | 0.41 | 0.029 | |
| MSMEG_1492 | MarR family transcriptional regulator | | | 0.27 | 0.012 | |
| MSMEG_3320 | LysR family transcriptional regulator, frameshifted | | | 0.48 | 0.014 | |
| MSMEG_3485 | Extracellular function sigma factor (RpoE1) | | | 0.28 | 0.044 | |
| MSMEG_4989 | Sensor histidine kinase | | | 0.44 | 0.012 | |
| MSMEG_4990 | Response regulator | | | 0.33 | 0.036 | |
| MSMEG_6253 | Fur family transcriptional regulator | | | 0.48 | 0.041 | |
|  |  | | |  |  | |
| **Hypotheticals – conserved** | | | |  |  | |
| MSMEG_0172 | Transmembrane protein | | | 0.46 | 0.024 | |
| MSMEG_0887 | Conserved hypothetical protein | | | 0.47 | 0.044 | |
| MSMEG_5215 | Conserved hypothetical protein | | | 0.37 | 0.041 | |
|  |  | | |  |  | |
| **Hypotheticals – nonconserved** | | | |  |  | |
| MSMEG_0584 | Hypothetical protein | | | 0.43 | 0.036 | |
| MSMEG_0979 | Hypothetical protein | | | 0.23 | 0.027 | |
| MSMEG_4499 | Hypothetical protein | | | 0.34 | 0.037 | |
| MSMEG_4961 | Hypothetical protein | | | 0.35 | 0.028 | |
| MSMEG_4992 | Hypothetical protein | | | 0.47 | 0.043 | |
| MSMEG_6254 | Hypothetical protein | | | 0.33 | 0.014 | |
| MSMEG_6676 | Transmembrane protein | | | 0.40 | 0.040 | |
| MSMEG_6739 | Hypothetical protein | | | 0.35 | 0.031 | |
|  |  | |  | | |  |
