# Supplementary material for: The Growth and Survival of Mycobacterium smegmatis Is Enhanced by Co-Metabolism of Atmospheric H2
Source: PLoS One. 2014 Jul 24;9(7):e103034. doi: 10.1371/journal.pone.0103034 (PMC4109961; doi:10.1371/journal.pone.0103034)
Supplement: Table S6 — List of intracellular and extracellular metabolites in Δ hyd 2 vs. wild-type cells. Metabolites were detected by gas chromatography-mass spectrometry (GC/MS). Values show the relative abundance of each metabolite detected in the samples (arbitrary units). Means were calculated from three biological replicates and five technical replicates for each strain and are shown to two significant figures. p values were determined using a Student’s T-test. Changes were classified as significant when p≤0.05 (Student’s T-test). (DOCX) [file pone.0103034.s009.docx]

|  | **Intracellular Samples** | | | **Extracellular Samples** | | |
| --- | --- | --- | --- | --- | --- | --- |
|  | **x̄_WT_** | **x̄_Δ_*_hyd_*_2_** | ***p*** | **x̄_WT_** | **x̄_Δ_*_hyd_*_2_** | ***P*** |
|  |  |  |  |  |  |  |
| L-Alanine-*d*_4_ | 1 | 1 | 1 | 1 | 1 | 1 |
| Aspartic acid | 1.1 | 1.2 | 0.976 | 0.018 | 0.017 | 0.904 |
| Azelaic acid | 0.046 | 0.032 | 0.058 | 0.0022 | 0.0023 | 0.896 |
| Benzoic acid | 0.73 | 0.98 | 0.333 | 0.0046 | 0.0053 | 0.298 |
| Decanoic acid | 9.3 | 8.3 | 0.171 | 0.046 | 0.49 | 0.597 |
| Dodecanoic acid | 2.3 | 2.1 | 0.449 | 0.032 | 0.034 | 0.555 |
| Glutamic acid | 0.19 | 0.38 | **0.046** | 0.044 | 0.042 | 0.840 |
| Lactic acid | 5.7 | 5.2 | 0.743 | 0.0045 | 0.041 | 0.251 |
| Leucine | 0.090 | 0.13 | **0.053** | 0.034 | 0.0033 | 0.905 |
| Levulinic acid | 0.034 | 0.034 | 0.970 | 0.0013 | 0.0018 | **0.047** |
| Myristic acid | 3.6 | 3.8 | 0.577 | 0.030 | 0.030 | 0.961 |
| Nicotinic acid | 0.16 | 0.13 | 0.158 | 0.0045 | 0.0060 | 0.068 |
| Octanoic acid | 84 | 81.5 | 0.574 | 0.0092 | 0.010 | 0.471 |
| Oleic acid | 6.8 | 9.2 | **0.006** | 0.026 | 0.029 | 0.533 |
| Palmitic acid | 19 | 19.9 | 0.520 | 0.030 | 0.025 | 0.624 |
| Quinic acid | 19 | 17.9 | 0.312 | 0.023 | 0.014 | 0.411 |
| Stearic acid | 5.0 | 5.0 | 0.891 | 0.059 | 0.065 | 0.662 |
| Suberic acid | 0.18 | 0.16 | 0.224 | 0.0033 | 0.0041 | 0.060 |
| Succinic acid | 2.8 | 3.2 | 0.208 | 0.012 | 0.0088 | 0.157 |
| *para*-Toluic acid | 0.69 | 0.68 | 0.991 | 0.0077 | 0.0088 | 0.244 |
| Tyrosine | 0.11 | 0.098 | 0.802 | 0.0013 | 0.0013 | 0.764 |
| *cis*-Vaccenic acid | 6.8 | 9.2 | **0.006** | 0.021 | 0.025 | 0.326 |
| Valine | 0.17 | 0.25 | **0.043** | 0.0060 | 0.0074 | 0.134 |
